# Underestimating Calorie Content When Healthy Foods Are Present: An Averaging Effect or a Reference-Dependent Anchoring Effect?

**DOI:** 10.1371/journal.pone.0071475

**Published:** 2013-08-14

**Authors:** Suzanna E. Forwood, Amy Ahern, Gareth J. Hollands, Paul C. Fletcher, Theresa M. Marteau

**Affiliations:** 1 Behaviour and Health Research Unit, Department for Public Health and Primary Care, University of Cambridge, Cambridge, United Kingdom; 2 Medical Research Council Human Nutrition Research, Cambridge, United Kingdom; 3 Department of Psychiatry, University of Cambridge, Cambridge, United Kingdom; University of Missouri-Kansas City, United States of America

## Abstract

**Objective:**

Previous studies have shown that estimations of the calorie content of an unhealthy main meal food tend to be lower when the food is shown alongside a healthy item (e.g. fruit or vegetables) than when shown alone. This effect has been called the negative calorie illusion and has been attributed to averaging the unhealthy (vice) and healthy (virtue) foods leading to increased perceived healthiness and reduced calorie estimates. The current study aimed to replicate and extend these findings to test the hypothesized mediating effect of ratings of healthiness of foods on calorie estimates.

**Methods:**

In three online studies, participants were invited to make calorie estimates of combinations of foods. Healthiness ratings of the food were also assessed.

**Results:**

The first two studies failed to replicate the negative calorie illusion. In a final study, the use of a reference food, closely following a procedure from a previously published study, did elicit a negative calorie illusion. No evidence was found for a mediating role of healthiness estimates.

**Conclusion:**

The negative calorie illusion appears to be a function of the contrast between a food being judged and a reference, supporting the hypothesis that the negative calorie illusion arises from the use of a reference-dependent anchoring and adjustment heuristic and not from an ‘averaging’ effect, as initially proposed. This finding is consistent with existing data on sequential calorie estimates, and highlights a significant impact of the order in which foods are viewed on how foods are evaluated.

## Introduction

Reducing calorie intake is a critical component of successful weight loss, and individuals needing to lose weight are frequently advised to do so by focusing on their calorie intake. This is facilitated when food packaging includes calorie-content information, but in the large number of situations when no calorie labeling is available, individuals must make an estimate of the calorie content of the food when making a choice, subject to imperfect knowledge and a range of perceptual biases.

A recent set of studies has highlighted one scenario where calorie content is systematically underestimated: Chernev [1], [2] asked participants to estimate the calorie content of a food, either alone or paired with an incidental side dish of healthy food (typically fruit or vegetables), and found that the inclusion of a small portion of healthier food elicited lower calorie estimates. For example, participants who viewed a cheeseburger paired with three celery sticks estimated it to contain fewer calories than those who viewed a cheeseburger alone, counter to the actual calorie content. This finding has been replicated by the same researchers using other high calorie main meals paired with fruit and vegetable side dishes [1], [2]. This error of judgment has been referred to as the “negative calorie illusion”, and paradoxically participants describing themselves as more concerned with their weight appear more prone to this bias, giving the largest underestimate – in some cases up to 100 calories [1].

The authors suggest it is the virtuous nature of the celery that drives this underestimate of the food’s energy content. The perceived healthiness of the virtuous food is averaged with that of the vice food, and leads to the meal as a whole being regarded as more healthy than the main meal alone. Because healthier foods are less likely to promote weight gain, they are also assumed to contain fewer calories [1], [2]. Thus, it is argued, those individuals who are more likely to invoke a vice/virtue categorization when assessing their food, such as those concerned with their weight, are also more likely to categorise a meal that includes a virtuous food as healthier than the meal alone, and therefore succumb to a larger negative calorie illusion.

This finding has important implications for public policy in the context of the current obesity epidemic. Incidental healthy foods, such as fruit and vegetables, are estimated to be used in 30% of advertisements for ‘energy dense nutrition poor’ foods [3]. Whether these illustrations impact on food purchasing and consumption is currently unknown, but the finding of a negative calorie illusion suggests that the growing trend to pair ‘energy-dense, nutrition-poor’ foods with incidental amounts of healthier foods could have undesirable consequences on food purchasing and consumption from a public health perspective by making consumers feel that they are eating fewer calories than they are.

The current study sought firstly to replicate the negative calorie illusion using methods that matched as closely as possible those used in previous studies [1], [2]. The importance of replication in psychology, particularly when studies reporting a phenomenon have only been conducted by one research team, is increasingly recognized [4], [5]. Alongside replication, the paper also sought to explore possible mechanisms underlying the negative calorie illusion. The principal explanation given in previous work proposes a healthiness assessment as mediating the calorie estimate. This has not been formally tested as none of the previous studies from Chernev and colleagues has also assessed perceptions of healthiness. The potential mediating effect of healthiness ratings on calorie estimates was therefore assessed in the current paper.

## Study 1

### Participants

301 participants, all residents of the United States of America and aged over 18 years, were recruited via an online panel (Mechanical Turk; https://requester.mturk.com/, [1], [6]). They received monetary compensation (US$2.10 for 15 minute testing) for taking part. Participants in the panel were mostly female (56.8%), with a mean age of 35 years and distributed across age groups as follows: 5% were 20 or younger, 38% were between 21 and 30, 29% were between 31 and 40, 18% were between 41 and 50, and the remaining 10% were over 51. All participants in this and the following studies gave informed consent via the website before being able to participate in the study. Ethical approval for all the following studies was provided by the University of Cambridge Psychology Research Ethics Committee (reference number: PRE 2011–57).

### Method

All testing took place online, with no face-to-face contact between participants and researchers. After consenting to take part in the study, participants were shown two meals and asked to estimate each meal’s calorie content. Participants were randomly allocated to one of three groups, with each group viewing the same two main meals. One group (n = 104) was shown the main meal items alone, one group (n = 99) was shown the same main meal items with a green healthy side dish, and the third group (n = 98) was shown the same main meal items with a red healthy side dish. The main meals used in this experiment were (the corresponding healthy options are given in parentheses): Cheeseburger (three celery sticks, raw carrot sticks), and meat lasagna (a green apple, small bunch of red grapes) (see Figure S1).

Each meal presentation included a visual image of a food and a written description. The choice of the stimuli is consistent with prior research [1], [2]. Under the image and description, participants were asked “About how many calories are in this food?” with a text box for them to type their answer as a positive integer.

Following calorie estimation, participants were presented with the same two foods again, and asked to rate the healthiness of the food on a five-point scale: “To what extent does this food fit with a healthy diet?” (Not at all/Not well/Somewhat/Very well/Perfectly).

It was decided to keep the phrasing of both the calorie estimate and the healthiness questions constant across all participants, even though some participants viewed images of one food item and others viewed images of two food items. Hence, both questions refer to ‘this food’, a term that directs participants to consider all the foods present in the image and is neither singular nor plural.

Following this, participants were asked to provide additional measures of weight concern using “To what extent are you concerned with managing your weight?” (Not at all concerned/slightly concerned/moderately concerned/extremely concerned) [1], and dietary restraint using the restraint scale of the TFEQ-R18 [7]. Participants self-reported their weight and height.

### Analysis

Analysis of variance was planned on calorie estimates and health estimates, with a main effect of the side dish and a main effect of main meal. If a significant effect of the side dish was found, the strength of the health estimates as mediators for the calorie estimates was then assessed.

The nature of calorie estimates made it likely that the data were not normally distributed. This is because calorie estimates cannot be negative, so there was a lower bound to the possible estimates –zero – but no upper bound. As a result, the range of possible values for under-estimates was smaller than the range of possible values for over-estimates, which generates a positively skewed distribution. The presence of a normal distribution is important for the use of the proposed statistical analyses, so if the data are found to be positively skewed, a log-transform may be required prior to analysis.

Previous studies using calorie estimates did not comment on the distribution of the data or the use of a transformation to correct for a non-normal distribution [1], [2]. In order to facilitate comparison with these studies, analyses on the raw data as well as the log-transformed data are reported if the data are found to be positively skewed.

### Results

All participants gave a calorie estimate and healthiness estimate for both the burger and the lasagna. Potential effects of order were first assessed using analysis of variance, with a two-factor model (order×main meal). No effect of order (F(1,102) = 0.369, p = 0.55) or interaction between order and main meal (F(1,102) = 1.712, p = 0.19) was found on calorie estimates. A similar analysis of healthiness scores indicated no effect of order (F(1,102) = 0.56, p = 0.46) or interaction between order and main meal (F(1,102) = 3.154, p = 0.053). All following analyses therefore combine data from both trials.

#### Analysis of raw data

**Figure 1:**
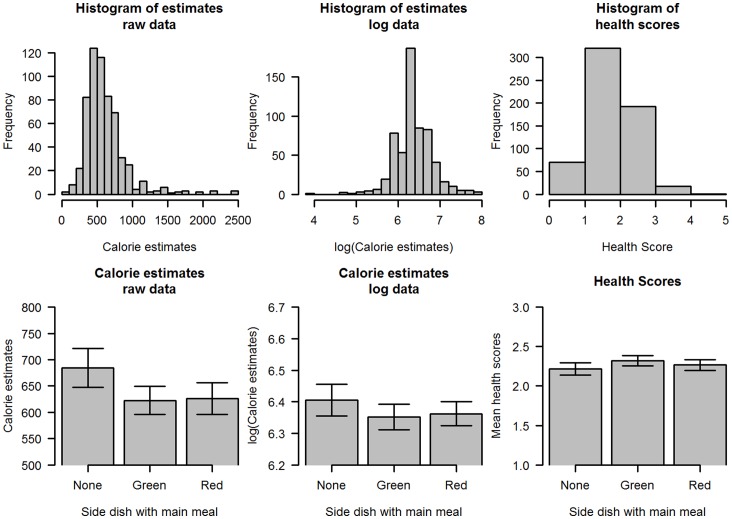
Calorie estimates from Study 1. Participants were asked to estimate to the calorie content of two mains presented alone, or the same two mains presented with a green healthy side, or the same two mains presented with a red healthy side. Histograms show the distribution of the calorie estimates for all three conditions combined. Since calorie estimates follow a positively skewed distribution, the natural log is taken, and the data re-plotted. Error bars indicate SEM.

Participants judged the main meal alone to have on average 678 calories, while the main with the green side was believed to have on average 616 calories and the main with the red side was believed to have 620 calories (see Figure 1). Analysis of variance of the raw calorie estimate data revealed no significant effect of main meal being assessed (F(1,596) = 2.364, p = 0.12) and a marginal non-significant effect of the side on the calorie estimates (F(2,596) = 2.432, p = 0.09).

#### Analysis of log-transformed data

Visual inspection of a histogram of the calorie estimates revealed that they are positively skewed (Figure 1). The conventional transformation for this non-normal distribution is to take the natural log of the raw data, for which a histogram reveals a normal distribution (Figure 1). Using this transform, participants judged the main meal alone to have on average 6.4 ln calories (or 604 calories), while the main with a green side was believed to have on average 6.35 ln calories (or 574 calories) and the main with the red side was believed to have 6.32 ln calories (or 580 calories). Analysis of variance of the natural log of calorie estimates revealed no significant effect of main meal being assessed (F(1,596) = 2.139, p = 0.14) and no significant effect of the side on the calorie estimates (F(2,596) = 0.86, p = 0.43).

#### Analysis of data with outliers removed

An alternative process to analyzing such data is to exclude data from participants that completed the task unusually quickly, suggesting poor engagement, and to exclude outliers (more than 2 standard deviations from the mean). Participants took on average 328 seconds (s.d. 217 second) and at least 128 seconds to complete the task, suggesting all engaged with the task to a similar extent. In terms of calorie estimates, three participants gave outlier estimates for the Burger (over 1213 calories), eight participants gave outlier estimates for the lasagna (over 1326 calories) and six participants gave outlier estimates for both foods. The remaining 284 participants judged the main meal alone to have on average 606 calories, while the main with the green side was believed to have on average 579 calories and the main with the red side was believed to have 586 calories. Analysis of variance of the raw calorie estimate data revealed no significant effect of main meal being assessed (F(1,562) = 2.678, p = 0.10) and no significant effect of the side on the calorie estimates (F(2,562) = 0.935, p = 0.39).

Analysis of variance of the healthiness rating of the food revealed a significant effect of main meal being assessed (F(1,596) = 21.658, p<0.001), with lasagna rated as more healthy than the cheeseburger and no significant effect of the side on healthiness ratings (F(2,596) = 1.127, p = 0.32).

Chernev [1] reported the largest calorie underestimates for participants expressing the highest levels of concern with their weight, which comprised 40/301 (13%) in this study. Our sample was therefore too small to power an analysis comparing participants expressing extremely high level of concern with all other participants.

Instead, weight concern as a between-participant factor with 5 levels was included as an additional factor in the analysis of variance of the natural log transformed data. No significant effect of weight concern were found either as a main effect (F(4,572) = 1.45, p = 0.22), or in an interaction with main meal (F(4,572) = 0.379, p = 0.84), with side dish (F(8,572) = 1.125, p = 0.34), or in a 3-way interaction (F(8,572) = 0.164, p = 0.99).

### Discussion

When raw calorie estimates were considered, the analysis showed no significant effect of the side dish on calories estimates although the data showed a pattern in agreement with previous findings [1], [2]. However, since this was skewed data, it should be treated with caution as the statistical tests assume the data to be normally distributed. When the data were normalized using a natural log transformation or by the removal of outlier calorie estimates this experiment failed to show any significant effect of the healthy side dish on calorie estimates. This would suggest that the pattern observed with the raw estimates data could have been the result of a small number of very high estimates distorting the group averages.

We found no increase in calorie estimates for the main with the side relative to the main meal alone despite more food being present. However, the actual calorie contents of these foods should be born in mind. A cheeseburger or a portion of lasagna will contain about 500 kilocalories, whereas a small apple or a small carrot (40 g) might only contain 20 to 60 kilocalories. Given the variance of calories estimates, the current study is unlikely to be able to detect an increase in calorie estimates of this magnitude.

It is of note that there were significant effects of the main dish on healthiness ratings but not on the calorie estimates, suggesting that health ratings did not necessarily determine calorie estimates, as has been assumed by Chernev and colleagues [1], [2]. As there were no significant effects of any of the dependent variables on calorie estimates, it was not possible to assess the mediating role of the healthiness rating.

This study failed to replicate the negative calorie illusion shown in previous studies [1], [2]. We retained as many aspects of the original studies as possible, though the failure to replicate might suggest important differences in the testing procedures used.

The number of participants in Study 1 (301 participants in 3 groups) is sufficient to detect the effect size observed in one of the previous demonstrations of the negative calorie illusion: Chernev et al [2] used 188 participants in 3 groups and found an effect of 0.47 standard deviations. But it is not powered to detect the effect size reported in a later replication [1] which involved many more participants (934 participants in two groups) and found a much smaller effect size of 0.23 standard deviations.

Another consideration is that the characteristics of participants in the current study differed from those in previous studies. Chernev [1] reports finding a larger negative calorie illusions in participants reporting higher levels of concern with their weight, and with such a minority showing extreme weight concern in Study 1, perhaps the make-up of the groups militated against finding the calorie illusion.

While the actual number of participants expressing each level of concern is not reported in Chernev [1], this can be approximated by assuming that all participants made the same number of estimates and using the total number of estimates given at each level of concern. Comparing this distribution with that from Study 1 using a Chi-Squared analysis does reveal a significantly different range of concern levels in the two studies (χ^2^(4) = 11.08, *p*<0.05), with Study 1 involving a lower proportion of individuals reporting high levels of concern, and a higher proportion of individual reporting a low level of concern. However, it should also be born in mind that the original demonstrations find a negative calorie illusion in calorie estimates across all participants, and not just those with high weight concern. It therefore seems unlikely that a difference in weight concern amongst study participants alone can explain the failure to replicate the negative calorie illusion.

## Study 2

Chernev and Gal argued that people perceived a meal combining a virtue and vice as being healthier than the vice alone [2]. They went on to argue that people rely on their evaluations of a meal’s overall healthiness to infer its calorie content. However, no evidence was put forward to support this link between perceived healthiness and calorie estimates. Given, this, we set up Study 2 in order to examine this possibility by broadening the healthiness range of foods being tested and to use the data generated to assess the mediating effect of healthiness ratings on calorie estimates.

Therefore, in Study 2, in addition to asking for calorie and healthiness estimates for a main meal alone, and a main meal with a healthy side dish, estimates were collected for the calorie content of the same main meal with an unhealthy side dish [2]. Study 2 also included a manipulation of the portion size of the side dish, so both side dishes were tested with a small portion size (as used in study 1) and a large portion size – roughly three times the size of that used in study 1.

### Participants

541 participants, all residents of United States of America and over 18 years, were recruited via an online panel (Mechanical Turk; https://requester.mturk.com/, [1], [6]). They received monetary compensation for taking part (US$2.10 for 15 minute testing). Participants in the panel were mostly female (58.4%), with a mean age of 34 years and were distributed across age groups as follows: 7% were 20 or younger, 40% were between 21 and 30, 30% were between 31 and 40, 12% were between 41 and 50, and the remaining 11% were over 51.

### Method

Participants were randomly allocated to one of five groups. The groups viewed the main meal item alone (n = 120), with a small portion of a healthy side (n = 105), with a large portion of a healthy side (n = 108), with a small portion of an unhealthy side (n = 97) or with a large portion of an unhealthy side (n = 111). The main meal used in this experiment was a cheeseburger, served either with celery sticks (the healthy side) or French fries (the unhealthy side), in either small or large portions (see Figure S1). The small portions were the same as those used in Study 1, and large portions were approximately three times the size.

The testing procedure was exactly that same as for Study 1, with all the same measures taken in the same manner.

### Analysis

Analyses of variance were planned on calorie and healthiness estimates. These were run separately for meals with the healthy and the unhealthy side and assessed a main effect of the side dish portion size on calorie estimates. Study 1 demonstrated the skewed nature of these calories estimates data, so for this and following studies, only the natural log transformed calorie estimates will be reported.

Where a significant effect of the side dish was found, the strength of the health estimates as mediators for the calorie estimates was assessed by running a linear regression model, both with and without healthiness estimates in the model. If the inclusion of healthiness estimates in the model reduces the effect size for the side dish, healthiness estimates can be said to mediate the effect.

### Results

**Figure 2:**
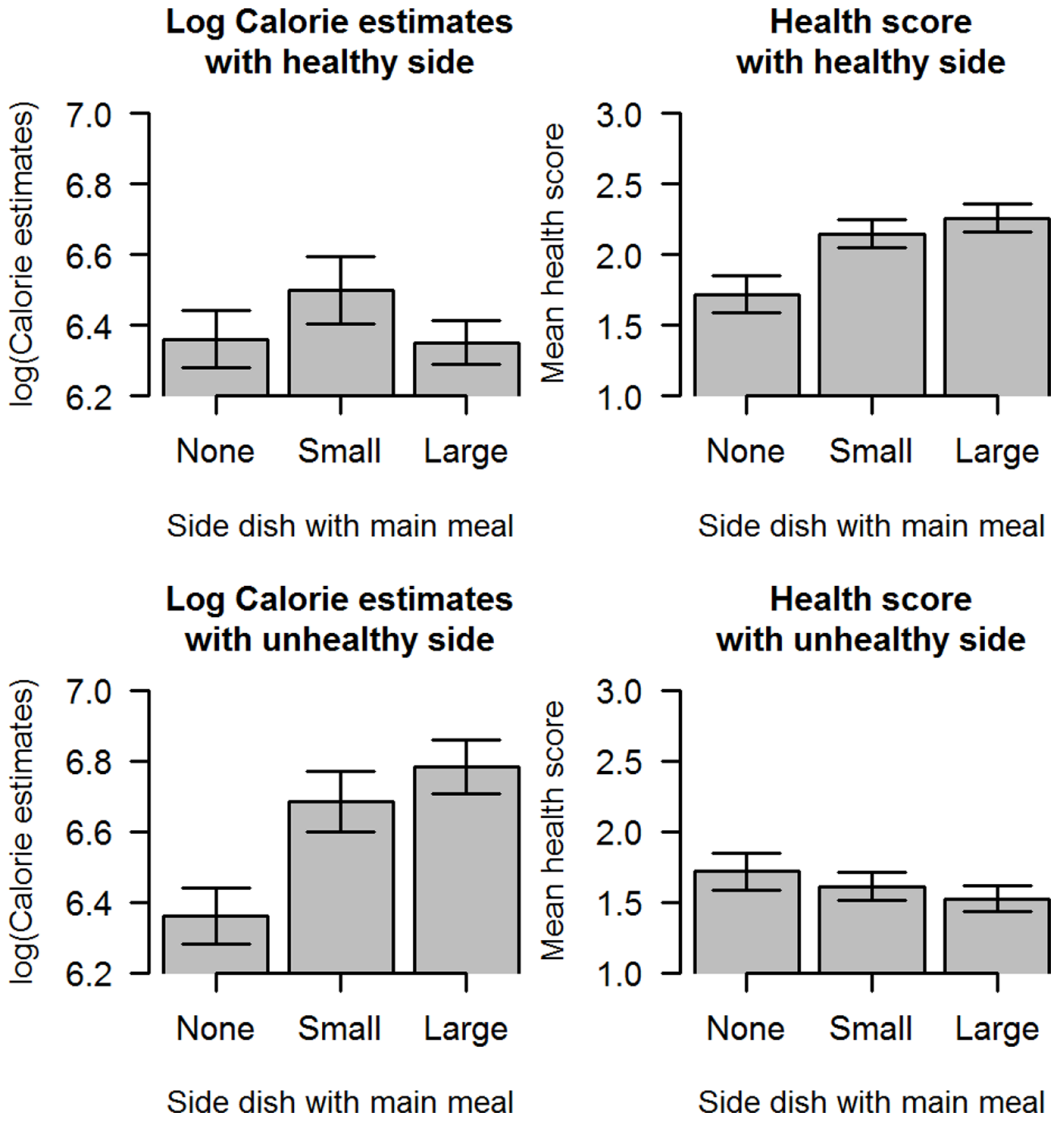
Calorie estimates and healthiness scores from Study 2. Participants were asked to estimate the calorie content of a main dish presented alone, or the same mains presented with a small portion of a healthy side, or a large portion of a healthy size, with a small portion of an unhealthy side or with a large portion of an unhealthy side. They were then asked to estimate the healthiness on a 5 point scale for the same two food images. Error bars indicate SEM.

Participants judged the main alone to have on average 6.35 ln calories (or 571 calories). The same main with a small portion of a healthy side was judged to have 6.50 ln calories (or 664 calories) and with a large portion of a healthy side to have 6.34 ln calories (or 567 calories). Analysis of variance of the log-transformed calorie estimates data with the healthy side revealed a non-significant effect of the healthy side on the calorie estimates (F(2,216) = 2.172, p = 0.12) (Figure **2**).

By contrast, participants judged the same main with a small portion of an unhealthy side to have 6.66 ln calories (or 781 calories) and with a large portion of an unhealthy side to have 6.73 ln calories (or 838 calories). Analysis of variance of the log-transformed calories estimates with the unhealthy side data revealed a significant effect of the unhealthy side on the calorie estimates (F(2,202) = 14.2,p<0.001) (Figure **2**). A post-hoc Tukey analysis of the main effect of side, which controls for multiple comparisons, revealed calorie estimates for both sized portions of the unhealthy side are significantly larger than estimates for the main without a side (both p<0.001), but estimates for calorie content of the main with a small side did not differ significantly from estimates with a large portion of an unhealthy side (p = 0.44). In other words, participants appeared to be sensitive to the addition of an unhealthy side, but insensitive to the portion size of that side when making their calorie estimates.

Since these data have shown a significant effect of the side dish on the calorie estimates, it was possible to assess the mediating effect of healthiness estimates on calorie estimates. As the small and large portions of side dishes did not yield significantly different calorie assessments, these were combined. The effect-size of side dish on calorie estimates in a linear regression model was compared with the effect-size when healthiness scores were included in the model. If healthiness scores acted as a mediator, the effect-size should fall with the inclusion of health scores.

This analysis revealed that the inclusion of healthiness estimates into the model actually increased the effect size. The effect of both side dishes on calorie estimates were to increase these estimates, the healthy side by 6.3% (p = 0.38) and the unhealthy side by 36.9% (p<0.001). Including healthiness scores into the model made little difference to the effect-size of side dish on calorie estimates and in both instances increased it – the healthy side to 16.3% (p = 0.03) and the unhealthy side to 37.3% (p<0.001). This finding suggests that healthiness scores, as measured by an explicit rating scale, were not acting as a mediator for calorie estimates, failing to support the hypothesis proposed by Chernev [2].

### Discussion

The findings from Study 2 demonstrate that participants estimating a main with an unhealthy side had more calories than the main alone. It is of interest to note that this increase in calorie estimates did not show sensitivity to portion size. This is in keeping with the literature on categorization of foods which shows that individuals are insensitive to quantity, even for nutrients when very low levels are essential for health while high levels are damaging, such as salt [8].

It is also of interest to note that participants did not seem to be using a judgment of healthiness as a mediator for making calorie estimates. This fails to support the main explanation of calorie estimate effect proposed by Chernev, that “people rely on their evaluations of a meal’s overall healthiness to infer its calorie content” [2, p44].

Study 2 provided a second failure to replicate the negative calorie illusion. This raises the question of how robust this finding is, and whether it can be explained by any protocol differences between the current study and previously published accounts [1], [2].

## Study 3

The results of studies 1 and 2 provoked an important and unanticipated question: why did we fail to demonstrate the previously-demonstrated negative calorie illusion? A number of possibilities could be considered, including differences in the characteristics of the stimuli used or the participants recruited. It should be noted that Study 2 did partially replicate the findings from one of Chernev’s studies in which an unhealthy side was added to a main [2, Experiment 1]. In that study, adding an unhealthy side increased calorie estimates by about 100 calories, while adding a healthy side reduced calorie estimates by about 100 calories relative to the estimate for the main alone. Yet, while this demonstrates the sensitivity of our paradigm to alterations in calorie estimation, the question of why we did not reproduce the negative calorie illusion remains.

One possibility, examined in study 3, is that the apparent discrepancy in findings emerges from a small but important difference between the Chernev studies and our own in terms of additional information presented to participants before completing the task. One of the original studies specified the use of a reference food [2, Experiment 1]. This is a main meal item labeled with its calorie content presented to the participants before they are asked to make their own calorie estimates, which was used by the experimenters to “reduce the variance resulting from people’s lack of precise calorie-content knowledge” [2, p40]. We were grateful to have our attention drawn to this in a personal communication with Chernev, in which it was confirmed that a reference item or other calorie content information was widely used in such studies, either before the current calorie estimate, or in previous tasks performed by same participants.

**Figure 3:**
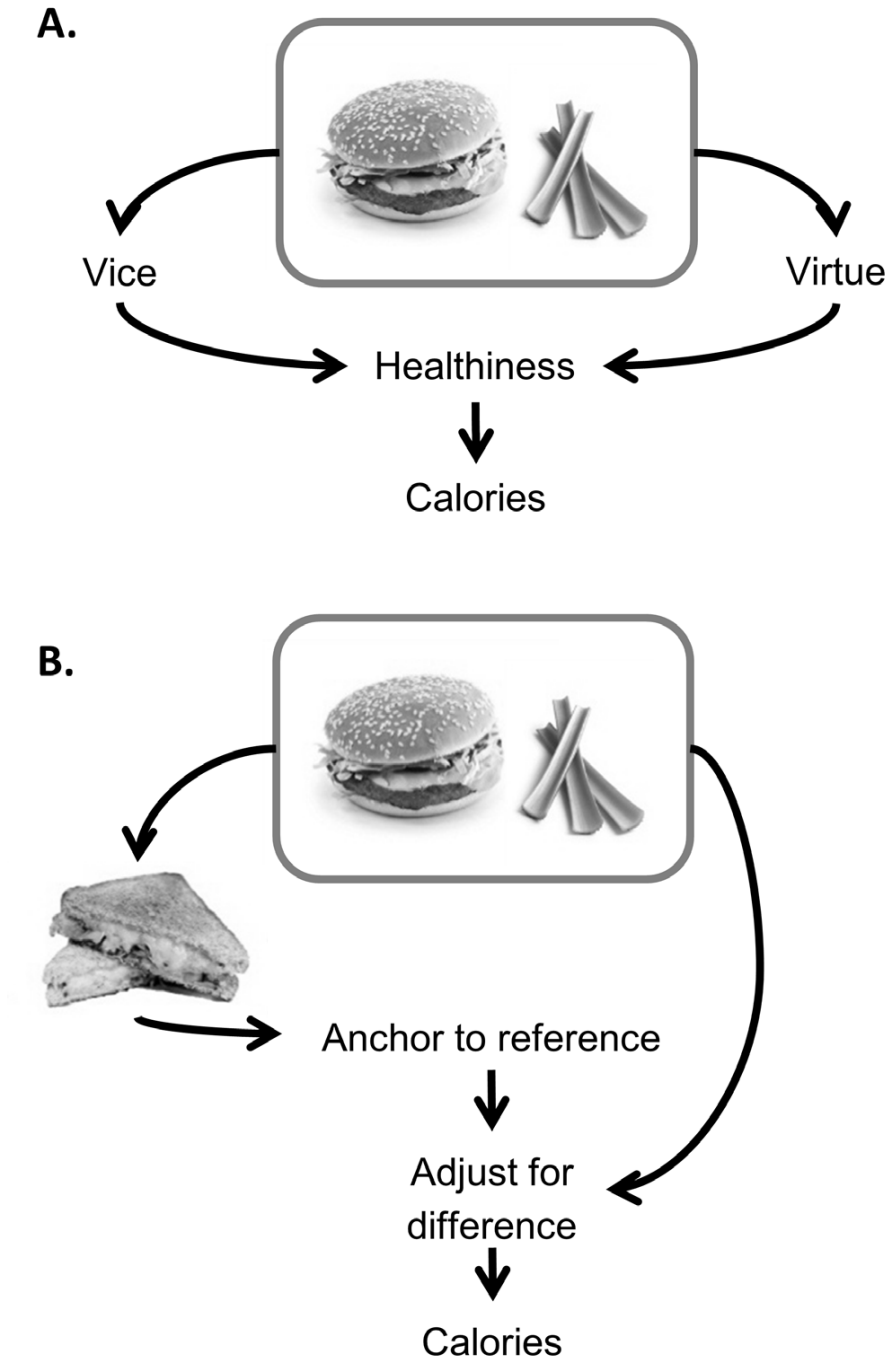
Competing hypotheses of how calories estimates are made. Left: Chernev proposes that individuals assess the vice or virtue nature of foods, and use this to estimate the healthiness of the combination of foods, which is then used to determine a calorie estimate [2]. Right: An anchoring and adjustment account [9], [10] proposes that individuals use the calories in the reference food as an anchor, adjusting this to capture the difference between the reference food and the target food.

The use of a reference food in previous Chernev studies that report the negative calorie illusion, and the fact that our first two studies, which did not use a reference point, did not elicit the negative calorie illusion, raises the interesting possibility that the presence of a reference food may have an important role in generating the negative calorie illusion. It is known that calorie estimates are inherently uncertain estimates, and in the absence of any accurate knowledge about the calorie content of food, participants may use the calorie content of the reference food as an anchor and then adjust this to estimate the content of the test food [9], [10](Figure **3**). If this anchoring and adjustment heuristic is indeed being used, it means that judgments about the test food are highly likely to be influenced by the nature of the reference food, and in particular by aspects of the test food that differ from the reference. In short, the estimate is likely to be reference-dependent. Thus, if the reference is a main meal item, and the test food is a main meal item with a healthier side dish, the side dish will be viewed or ‘coded’ as a gain. The gain of a conspicuously healthy food item will therefore drive calorie estimates lower than they would have been had the test food been a main meal item alone (Table 1, Groups 1 and 2).

**Table 1:** Competing predictions of the conditions under which a negative calorie illusion (NCI) will occur.

Study	Group	Reference food	Test food	Foodcategory	Health averagingprediction	Adjustmentdirection	Anchoring prediction
1 & 2	Main alone	–	A	Vice	–	None	–
	Main+healthy side	–	Ax	Vice+virtue	NCI	None	–
3	Group 1	B–500 cal	A	Vice	–	None	–
	Group 2	B–500 cal	Ax	Vice+virtue	NCI	↓ (New side)	NCI
	Group 3	By–500 cal	A	Vice	–	↓ (Less food)	NCI
	Group 4	By–500 cal	Ax	Vice+virtue	NCI	None	–

The letters A, B, x and y represent food. Main meals vice items, such as a cheeseburger, are represented by capital letters A and B. Healthy side dishes, such as celery sticks, are represented by lower-case letters x and y.

By contrast, Chernev proposes that all foods are categorized according to a good/bad dichotomy of healthy or unhealthy (vices or virtues), with meals combining vices with virtues perceived as more healthy than a vice alone. This overall evaluation of the meal’s healthiness is then used to make the calorie estimates [2] (Figure **3**). By this account, the negative calorie estimate will always be present when a virtue food is included in the meal to be assessed.

If calorie estimates are driven by reference-dependent anchoring and adjustment, participants will be strongly influenced by attributes on which the reference and test food differ as these will be coded as losses or gains, and use calorie knowledge of these attributes alone to adjust the calorie information from the reference. Calorie estimates are therefore very susceptible to the nature of the material presented prior to making the calorie judgment, i.e. the reference food, for subsequent judgments.

Thus a reference-dependent anchoring account is able to explain the differences between the findings from Studies 1 and 2, and those from previously published research [1], [2]. By not using a reference food at all, the healthy side dish was not coded as a gain in Studies 1 and 2, and did not reduce calorie estimates of a main meal with a healthy side dish relative to estimates of the main meal alone (Table 1). By contrast if the procedure used in Studies 1 and 2 is modified to include a reference food without a healthy side dish, as previously used by Chernev, an anchoring account would predict that a negative calorie illusion should emerge (Table 1, Group 1&2).

Since the reference-dependent anchoring account places so much weight on the content of the reference food, which the Chernev account does not, the two hypotheses are empirically discriminable. One instance when the two hypotheses generate opposing predictions is when the reference food includes a small healthy side (Table 1, Groups 3 & 4). The Chernev account, based on averaging healthiness, would always predict a negative calorie illusion when the calorie content of a main with a healthy side is estimated relative to a main without a healthy side, as long as the reference food is held constant. However the reference-dependent anchoring account would predict that the use of a reference food with a healthy side will prevent the healthy side dish in the test food being coded as a gain. In contrast it would predict that when a reference food with a healthy side is presented prior to a test food with no side, the side dish will be coded as a loss and should result in a lower calorie estimate than for a test food that includes a healthy side.

Study 3 tested the predictions of a reference-dependent anchoring hypothesis against Chernev’s hypothesis for the negative calorie illusion. A 2×2 design was be used, with test food (with/without healthy side) and reference food (with/without healthy side) as the variables of interest. The main predictions of Chernev’s hypothesis were a main effect of test food side dish, and no interaction between test food and reference food, since it is argued that the negative calorie illusion is a function of the combination present in the test food alone. By contrast the main predictions of the anchoring hypothesis were an interaction between test food and reference food, and no main effect of test food, as it is the contrast between the reference food and the test food that are hypothesized to drive calorie estimates.

### Participants

430 participants, all residents of the United States of America and over 18 years, were recruited via an online panel (Mechanical Turk; https://requester.mturk.com/, [1], [6]). They received monetary compensation for taking part (US$1.10 for 8 minute testing). Participants in the panel were mostly female (57.0%), with an average age of 35 years and were distributed across age groups as follows: 5% were 20 or younger, 41% were between 21 and 30, 22% were between 31 and 40, 16% were between 41 and 50, and the remaining 16% were over 51.

### Method

After consenting to take part in the study, participants were randomly allocated to one of four groups in a 2 by 2 design. Participants were shown a reference meal, which was always labeled as containing 500 calories, followed by a test meal and asked to estimate the calorie content of the test meal. Two groups were shown a main meal as the reference, followed by a test food of a main meal item alone (n = 103), or with a small portion of healthy side (n = 110). The other two groups were shown a main meal with a healthy side as the reference, followed by a test food of a main meal item alone (n = 106) or with a small portion of healthy side (n = 111). The reference food used in this study was a simple hamburger (with carrot batons), and the test food used in this study was a cheeseburger (with celery sticks) (see Figure S3 images used).

The remaining testing procedure was exactly that same as for Studies 1 and 2, with all the same measures taken in the same manner.

### Analysis

Analyses of variance were planned on calorie estimates including a main effect of the healthy side in the test food, a main effect of the healthy side in the reference food, and an interaction between the presence of a side in the test food with the presence of a side in the reference food. As for Study 2, analyses for only natural log transformed calorie estimates will be reported.

### Results

**Figure 4:**
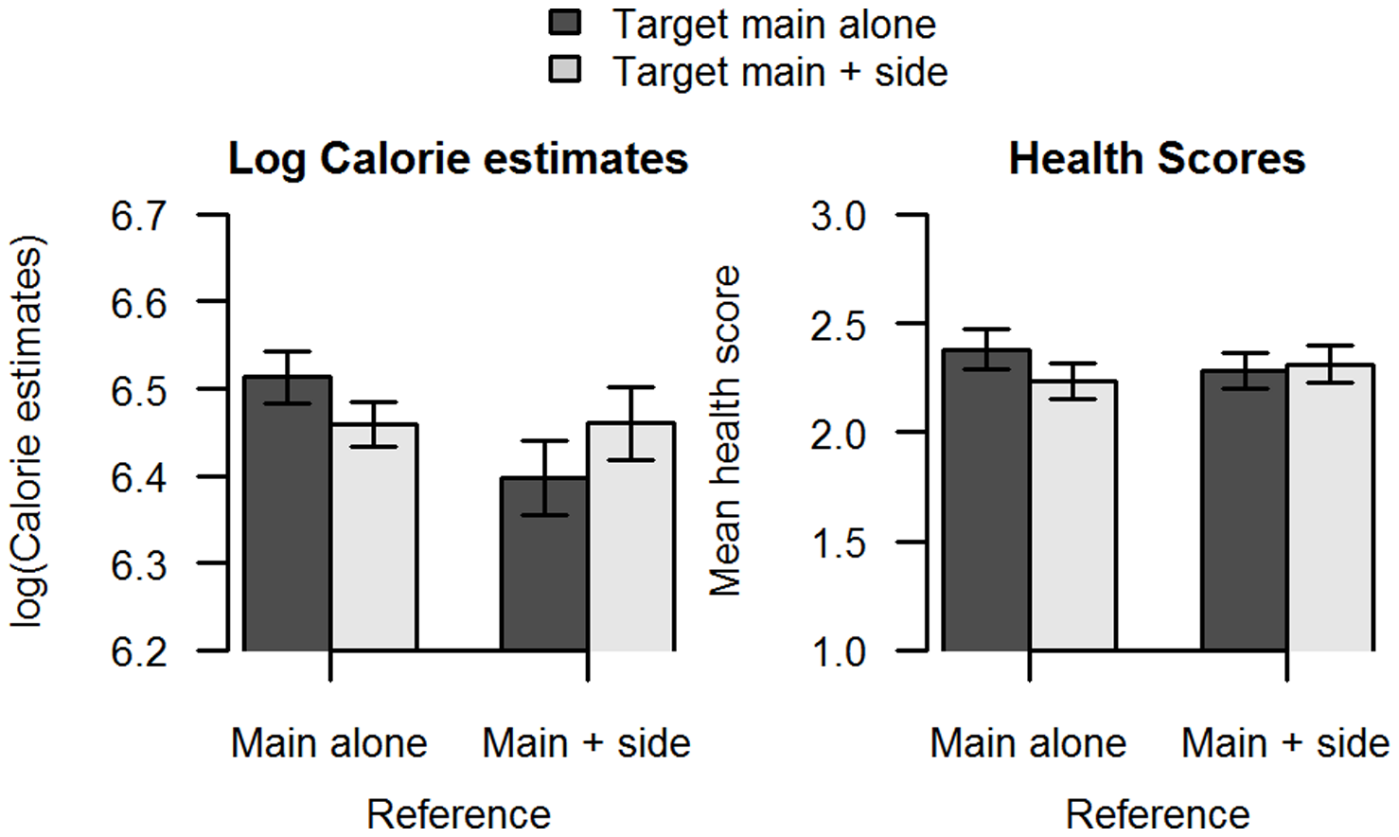
Calorie estimates and healthiness scores from Study 3. Participants were asked to estimate the calorie content of a main dish presented alone, or with a small portion of a healthy side. In addition, before making their calories estimate, participants were shown a reference food labeled as 500 calories. This was either a main dish alone or a main dish with a healthy side. They were then asked to estimate the healthiness on a 5 point scale for the same two food images. Error bars indicate SEM.

Participants shown the reference with no side judged the main alone to have on average 6.51 ln calories (or 674 calories), while the main meal with a healthy side dish was judged fewer calories with on average 6.46 ln calories (or 638 calories). By contrast, participants shown the reference that included a healthy side judged the main alone to have on average 6.40 ln calories (or 600 calories) while the main with a healthy side was judged to have more calories with on average 6.46 ln calories (or 639 calories). Analysis of the log-transformed calorie estimates revealed no significant effect of the healthy side in the test food on calorie estimates (F(1,431) = 0.042, p = 0.84), a significant effect of healthy side in the reference food on calorie estimates (F(1,431) = 4.63, p = 0.03), and also a significant interaction between a healthy side in the reference and a healthy side in the test food on calorie estimates (F(1,431) = 5.24, p = 0.02) (Figure **4**). Looking at the data reveals that when the reference food was a main meal alone, the test food with a healthy side was estimated to have fewer calories than the test food alone. By contrast, when the reference food included a healthy side, the test food with a healthy side was estimated to have more calories that the test food alone.

Analysis of variance of the health rating data reveal no significant effect of the healthy side in the test food on health estimates (F(1,431) = 0.83, p = 0.36), no significant effect of healthy side in the reference food on health estimates (F(1,431) = 0.01, p = 0.94), no significant effect of concern on health estimates (F(1,431) = 2.02, p = 0.16).

### Discussion

These data provide support for the reference-dependent anchoring and adjustment account of the negative calorie illusion as it was found that the nature of the reference food significantly interacted with the impact of a healthy side dish on calorie estimates. Using a reference food, or anchor, with no incidental healthy food generated the negative calorie illusion as seen in previous studies [1], [2]: a main with a healthy side was estimated to have fewer calories than a main alone. By contrast, using a reference food that included a healthy side gave the opposite pattern of calorie estimates: the main with a healthy side was estimated to have more calories than the main alone.

In addition to providing some insight into the mechanism behind the negative calorie illusion, these findings suggest that the reason for a failure to find a negative calorie illusion in Studies 1 and 2 is due to the absence of any reference or anchoring information. In the absence of such information, there would be no altered salience to any components of the meal, so it can be presumed that those data represented a cleaner account of the influence of incidental healthy foods on calorie estimates.

## General Discussion

Studies 1 and 2 did not replicate the negative calorie illusion. In addition, Study 2 sought evidence that calorie estimates were mediated by the perceived healthiness of the food, as proposed by Chernev [1], [2] but looking at the statistical effect size in a model without, and then with healthiness estimate data. These data show no evidence of a mediating role for health estimates, as measured by an explicit rating scale, on calorie estimates.

Study 3 tested an alternative hypothesis for the negative calorie illusion, namely a reference- dependent anchoring and adjustment account [9], [10]. Calorie estimates were collected for a main with and without a healthy side subsequent to viewing a reference food either with or without a healthy side, and it was found that the magnitude of the calorie estimates were driven as much by the reference food as by the test food, with the contrast between the two being central to any biases of judgment.

### Theoretical Implications

The current studies taken as a whole support an alternative explanation for the negative calorie illusion to that proposed by Chernev and thereby specify more precisely the contexts in which the illusion is most likely to occur [1], [2]. Chernev’s account of his original findings was to suggest that participants make healthiness estimates for the combination of food presented to them – be it entirely vice foods or a combination of virtue and vice foods. These healthiness estimates are then proposed to drive calorie estimates. Study 2 was able to test the role of perceived healthiness of the food combinations in mediating calorie estimates by looking at effect sizes in regression models both with and without health estimates as a covariate. The data from this study provide no evidence for a mediating effect of health estimates on calorie estimates. Data from Studies 1 and 3 also support this observation, as both found different patterns of effects for health estimates and for calorie estimates.

Instead, the findings from Study 3 suggest that the negative calorie illusion results from the use of a reference-dependent anchoring and adjustment heuristic to make estimates about the test food relative to the reference food, whereby the difference between the reference and the test food is coded as a loss or a gain, and thereby influences calorie estimates. This finding is consistent with existing data on sequential calorie estimates [11], where it has been shown in a number of studies that the order in which foods are presented can quite substantially bias calorie estimates. If vices are judged after virtues, they are perceived to have more calories than if they are judged after another vice; in other words the calorie estimate is biased in favor the aspects of the food that differ in a sequence of foods.

### Real-world Implications

Calorie estimates are a simple measure of participant’s perception of foods; however they almost certainly do not reflect actual factual knowledge about a food’s calorie content. It is not currently known whether calorie estimates are related to the expected satiety for a food, or anticipated tastiness. The data from the current studies fail to show that calorie estimates are derived directly from the healthiness ratings of foods. Other studies have shown that calorie estimates are influenced by the restaurant from which a food is purchased [12], as well as the order in which foods are presented [current study, 11], very much supporting the contextually sensitive nature of calorie estimates. And there is some evidence that erroneous calorie estimates alter portion size selection [13] and that lower calorie estimates for a main meal item have been shown to alter selection for drinks and side dishes [12].

Based on the current data, a negative calorie illusion is unlikely to be driving systematic failures in calorie estimations when incidental “healthy foods”, such as fruit and vegetables, are viewed alongside energy dense nutrition poor foods in advertisements or food labels. Foods would need to be viewed in a pre-determined sequence for systematic errors in real-world instances of calorie estimates. A couple of examples when this might occur are when food items are viewed in a meal with courses (starter, main, dessert) or when foods are seen in a specified order as they are positioned on a food menu or within the pathway around a supermarket from the entrance to the checkout tills. Further experimentation within these two particular contexts would be needed to see if biases are present, and if so, what influence they have on preference and consumption of food.

### Replication in Science

The current study provides an attempt to replicate a published finding, along with additional data that adds to our understanding of how participants generate calorie estimates. Alongside the merits of understanding calorie estimates, this study echoes some of the issues of replications in scientific research in general [4], [14], and more recently in psychological research in particular [5], [15]. Two prominent sets of psychological experiments have highlighted the difficulty in replicating psychological research using the same methods as described in the original publication, as Studies 1 and 2 attempted to do, one of these concerning priming effects [16], [17] and the other precognition [18], [19]. However, unlike these examples, the current study provides both replication and extension by identifying an experimental variable that was given little or no mention in the original methods, the use of a reference food, which appears to be of critical importance in eliciting the phenomenon of calorie underestimates. The existence of important variables overlooked in the original studies may serve to explain both why different research teams fail to replicate a published finding while apparently using the same methods as reported in the publications, and what the mechanism underpinning the phenomenon in question is. While some will continue to debate whether or not a particular phenomenon is a statistical anomaly or a true finding, more tractable advances are more likely to result from determining under what conditions and therefore by what mechanisms a phenomenon is revealed.

### Limitations

One key limitation of both these studies, and the original studies which these are replicating [1], [2], [11] is that no real calorie information is presented as the reference or included in the analysis. This means that it is not possible to determine under what circumstances individuals are accurate in their estimates, or whether the estimates are over-estimates or under-estimates of reality. Some would note that in study 1 and 2 we did not observe calorie estimates being greater when the healthy side dish is added to the main meal item, something that could be argued to be counter-factual. However, the healthy side dishes consisted of fruit or vegetables, and the actual calorie content of these is likely to be very low (e.g. a 40 g portion of carrot contains 59 kcal and a 40 g portion of celery contains 13 kcal) by comparison with a burger (McDonalds BigMac –550 kcal), making any veridical assessments hard to detect.

A further replication of the current study that would be of considerable interest is in exploring whether calorie estimates accurately reflect the true energy content of the food. Such a study would need more controlled photographic stimuli, including cues that allow participants to more accurately gauge the size of the food portions, such as plates, knives and forks, as well as known calorie content for the food, and as such might be better conducted in person rather than online using images.

### Conclusion

The current set of studies sought to replicate the negative calorie illusion. The results show first, that the absence of a reference food obliterates the negative calorie illusion (studies 1 and 2) and second, that the presence of a reference food reinstates it and demonstrates that it is not a ‘heathiness averaging effect’ as was originally proposed [2], but rather the consequence of the use of an anchoring and adjustment heuristic [9], [10].

Calorie estimates are an interesting index of our perceptions of the properties of food. They are easily captured and the current studies, together with existing research on calorie estimates, demonstrate the extent to which they are influenced by the context within which a food is presented. Their variation may be a useful measure of our perception of the food we are eating and a target for intervention to alter the choices we make and reduce energy consumption.

## Supporting Information

Figure S1 Supplementary Material showing the images of food shown to participants in each experimental group. A: Food images used in study 1: Participants saw one of image of each main dish. B: Food images used in study 2: participants viewed only one of the food images. C: Food images used in study 3: participants viewed one of reference foods followed by one of the target foods.
(PDF)
