# Supplementary figures and images for: Underestimating Calorie Content When Healthy Foods Are Present: An Averaging Effect or a Reference-Dependent Anchoring Effect?
Source: PLoS One. 2013 Aug 14;8(8):e71475. doi: 10.1371/journal.pone.0071475 (PMC3743811; doi:10.1371/journal.pone.0071475)

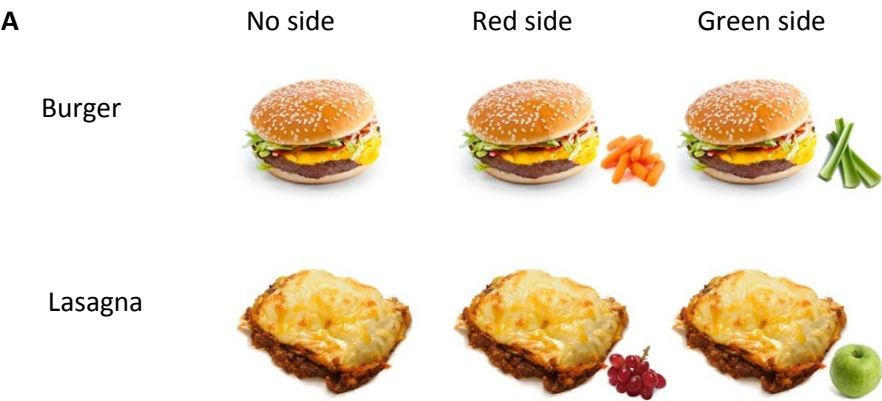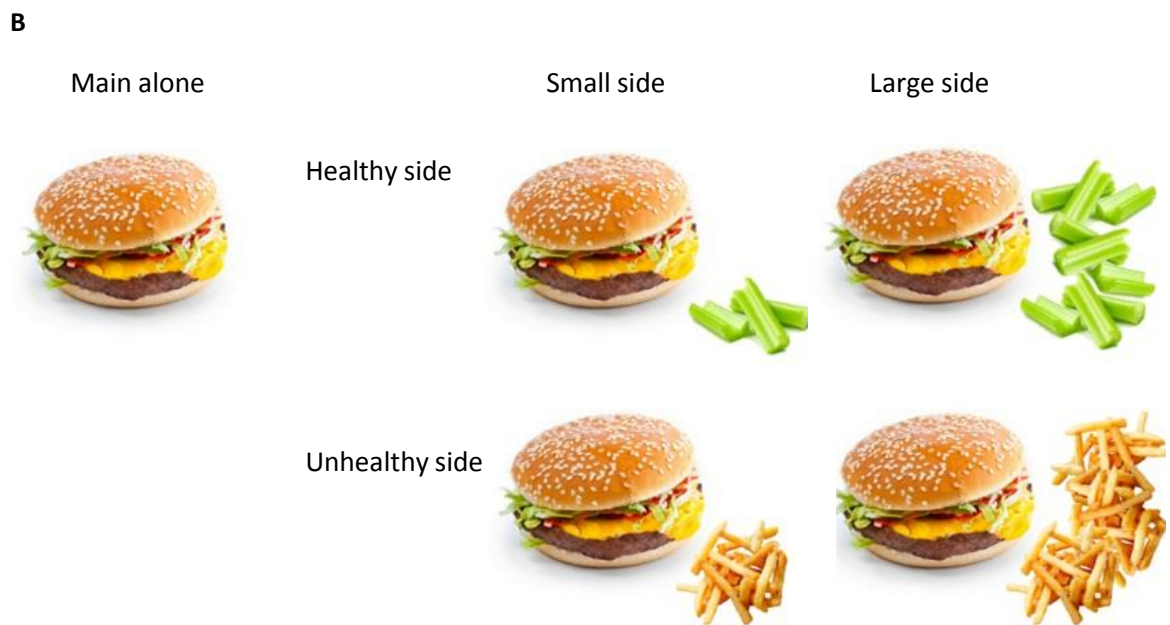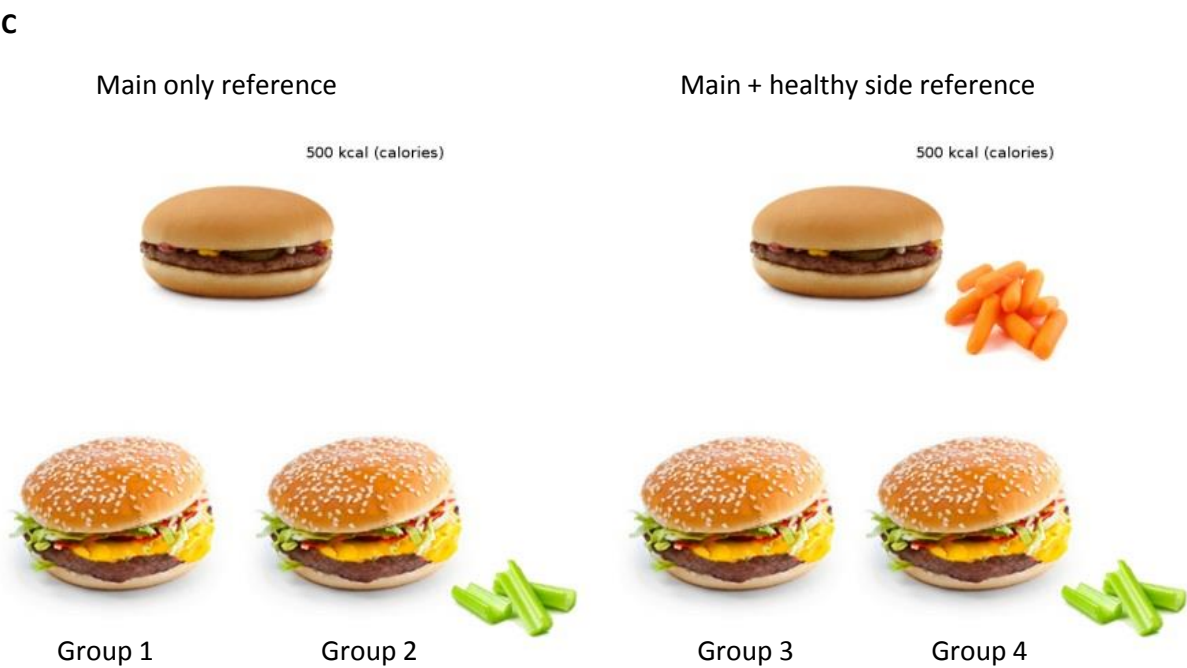

Supplement: Figure S1 — Supplementary Material showing the images of food shown to participants in each experimental group. A: Food images used in study 1: Participants saw one of image of each main dish. B: Food images used in study 2: participants viewed only one of the food images. C: Food images used in study 3: participants viewed one of reference foods followed by one of the target foods. (PDF) [file pone.0071475.s001.pdf]
